# Shifts in Southeastern Bat Communities Driven by Landscape Composition Rather Than Ecological Release

**DOI:** 10.1002/ece3.74129

**Published:** 2026-07-31

**Authors:** Dakota J. Van Parys, Sarah C. Williams, Catherine G. Haase

**Affiliations:** ^1^ Center of Excellence for Field Biology Austin Peay State University Clarksville Tennessee USA; ^2^ Department of Biology Austin Peay State University Clarksville Tennessee USA; ^3^ Environmental Division US Army Fort Campbell Fort Campbell Kentucky USA

**Keywords:** ecological niche, ecological release, landscape composition, landscape metrics, southeastern bat communities, white‐nose syndrome

## Abstract

Ecological niches are shaped by both abiotic conditions and biotic interactions and shifts in species composition, such as the loss of competitors or habitat availability, can trigger cascading changes in community dynamics. Emerging infectious diseases offer natural experiments to observe such shifts in real time. Since its detection in 2006, white‐nose syndrome (WNS), caused by the fungal pathogen *Pseudogymnoascus destructans*, has decimated several North American bat species, with mortality closely tied to species‐specific susceptibility. Yet, disease alone does not account for ongoing changes in bat populations. Habitat availability, landscape composition, and landscape configuration may also strongly influence bat capture rates and community dynamics following WNS invasion. We evaluated long‐term changes in bat capture rates on Fort Campbell Military Installation, Tennessee and Kentucky, USA, to determine whether variation in capture rates of non‐susceptible species was associated with declines in WNS‐susceptible species or with landscape composition and configuration. Using 30 years of mist‐net capture data (1998–2023), we modeled capture rates of four non‐susceptible species ( *Eptesicus fuscus* , *Lasiurus borealis* , *Myotis grisescens* , and *Nycticeius humeralis* ) at both installation and site spatial scales. Candidate models included capture rates of WNS‐susceptible species, forest composition and configuration metrics, temperature, and precipitation. Installation‐level analyses were conducted using generalized linear models, whereas site‐level analyses used zero‐inflated negative binomial models to account for excess zeros and overdispersion. Results reveal that some non‐susceptible species exhibited post‐WNS increases, but these trends were species‐ and scale‐dependent and not universally consistent with ecological release. Furthermore, landscape features, particularly forest cover and patchiness, significantly explained variation in capture rates across space and time for certain species. These findings indicate that bat community dynamics at Fort Campbell reflect the combined influence of disease and habitat availability rather than a singular driver. This study underscores the complexity of post‐disease community restructuring and highlights the need to consider interacting ecological stressors when assessing wildlife responses to disturbance and emphasizes the need for integrated approaches to wildlife conservation.

## Graphical abstract

Using 25 years of capture data from Fort Campbell Military Installation, we evaluated whether white‐nose syndrome (WNS)‐driven declines in susceptible bats were followed by increases in non‐susceptible species (ecological release) versus shifts driven by land use change, at the installation and site specific spatial scales. Susceptible species capture rates fell by ~69% while non‐susceptible species capture rates rose ~39%, but responses were species & scale dependent and inconsistent with classic competitive release (relationships were positive rather than negative). Rather, landscape metrics (e.g., forest patchiness, clumpiness, edge density) explained substantial variation for several species, indicating post‐WNS community dynamics reflect interacting effects of disease and habitat change rather than a single driver.

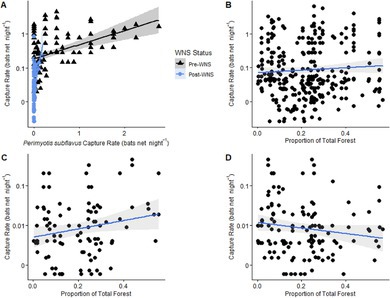

## 1 Introduction

Hutchinson (1959) described an ecological niche as an “n‐dimensional hypervolume,” stating that multiple variables explain and constrain when and where a species can exist. The niche can be broken down into two parts: the fundamental niche and the realized niche. The fundamental niche is defined as the range of environmental conditions, such as temperature, humidity, soil salinity, etc., that a species can potentially exist in, whereas the realized niche is the area that a species actually occupies as a consequence of competition, predation, and available food sources (Hutchinson 1959). Given that the realized niche is shaped by complex biotic interactions, it is inherently flexible and can shift over time; it reflects the balance between intraspecific competition, which promotes niche diversification, and interspecific competition, which constrains niche breadth (Grant and Price 1981; Taper and Chase 1985). The realized niche may further shift in response to changing ecological conditions, such as the loss of predators (Beschta and Ripple 2006) or competitors (Thalken et al. 2018), allowing a species to expand into areas of its fundamental niche that were previously unavailable. Such an expansion of the realized niche in response to reduced ecological pressures is known as an ecological release (Roughgarden 1972).

Human impacts on ecosystems, such as species extirpation through overexploitation, habitat destruction, or disease, can create natural experiments to study ecological release. The loss of competitors or predators in these altered environments allows researchers to observe how a species may expand their realized niche through new resource availability or the release of other biological constraints. The introduction of the invasive fungus *Pseudogymnoascus destructans* (*Pd*) provides an opportunity to study how variance in susceptibility to pathogens can result in an ecological release. Since 2006, multiple hibernating bat species in North America have been experiencing mass mortalities due to white‐nose syndrome (WNS), a disease caused by *Pd* (Blehert et al. 2009). *Pd* is a cold‐loving fungus that can persist in the substrate of hibernacula, such as soil, rock surfaces, and cave sediments, acting as an environmental reservoir; thus, *Pd* can survive without the presence of a host, making it a prolific pathogen. When on a host, *Pd* invades the skin tissue of the rostrum and wings of hibernating bats (Gargas et al. 2009), causing severe damage resulting in excessive evaporative water loss (Cryan et al. 2010). This pathology results in disruption to the torpor‐arousal behavior of hibernating bats (Reeder et al. 2012), increasing fat consumption and leading to mortality (McGuire et al. 2017; Turner et al. 2015).

Not all North American bat species have experienced severe mortality from WNS; however, allowing WNS susceptibility to be classified based on the fungal loads each species exhibits when exposed to *Pd* (Langwig et al. 2016). The species in the high susceptibility range are the little brown ( *Myotis lucifugus* ; Hoyt et al. 2018), northern long‐eared ( *Myotis septentrionalis* ; Hoyt et al. 2018), Indiana ( *Myotis sodalis* ; Langwig et al. 2012), and tricolored bats ( *Perimyotis subflavus* ; Jackson et al. 2022). The high susceptibility of these species is due to increased pathogen exposure from sociability during hibernation, lower fat stores given their smaller body masses, and selection of roost areas of caves where the humidity and temperature are more optimal for *Pd* growth (Bernard and McCracken 2017; Haase et al. 2019, 2021; Johnson et al. 2016). Big brown ( *Eptesicus fuscus* ; Bombaci et al. 2021), eastern small‐footed ( *Myotis leibii* ; Bombaci et al. 2021), and gray bats ( *Myotis grisescens* ; Powers et al. 2016) are considered to be in the low susceptibility range based on their low sociality during hibernation, larger body mass, and selection of roosts in colder and drier portions of caves with little to no *Pd* growth (Bernard et al. 2017; Geiser 2004; Haase et al. 2021; Langwig et al. 2012; Powers et al. 2016). Some bats have shown no susceptibility to WNS, such as the evening ( *Nycticeius humeralis* ), hoary ( *Lasiurus cinereus* ), silver‐haired ( *Lasionycteris noctivagans* ), and eastern red bats ( *Lasiurus borealis* ; Bombaci et al. 2021). These bats are either solely tree‐roosters ( *L. borealis* ), roost in areas of caves that provide unsuitable growth conditions for *Pd* ( *N. humeralis* , *L. noctivagans* ), or are migratory and do not hibernate ( *L. cinereus* ) (Cryan 2003; Limpert et al. 2007; Mager and Nelson 2001; Mattson et al. 1996).

Although WNS has caused massive die‐offs in many affected bat species, numerous cases of increased activity and abundance of non‐susceptible bat species have been documented across North America since WNS emergence, possibly due to the release from competition for foraging and roosting resources. The collapse of the *M. lucifugus* population in Fort Drum, New York, led to a spatial and temporal shift in foraging activity for five of the other six bat species in the local area (Ford et al. 2011; Jachowski et al. 2014). In southern Ontario, a 28% increase in foraging activity was observed in *E. fuscus* compared to an 82% decrease in *M. lucifugus* foraging when comparing pre‐WNS invasion to post‐invasion years (Morningstar et al. 2019). A 276% increase in *E. fuscus* activity was correlated with a 100% reduction in foraging activity in southeastern Pennsylvania for *M. lucifugus* and *M. septentrionalis* due to WNS (Hauer et al. 2019). Prior to the introduction of *Pd*, *M. septentrionalis* was the most common species to be captured at Mammoth Cave National Park in Kentucky, with *N. humeralis* being the least common (Lacki et al. 2015). After WNS invasion, *M. septentrionalis* suffered a drastic decline in abundance, with *N. humeralis* experiencing the largest increase in abundance, and this is believed to be due to the availability of previously unavailable roosts (Thalken et al. 2018).

While WNS has contributed to population declines in several bat species, it is not the sole threat to North American bat populations. As a result, population changes in non‐susceptible bat species may not follow expected patterns if other environmental or ecological factors also shape their abundance. Across the globe, the amount, configuration, and quality of habitat have been shown to significantly influence bat diversity and abundance (Mehr et al. 2011; Mendes and Srbek‐Araujo 2020; Meyer et al. 2016; Pretorius et al. 2021). In North America specifically, Adams et al. (2024) found that 53% of bat species are either already listed or are at risk of being listed as threatened or endangered, with habitat cited as a major driver of these trends (Thompson et al. 2017). For most North American insectivorous bats, the extent of forest cover and its complexity can influence foraging and roosting behavior (Dixon 2012). In addition to forest availability, the configuration of habitat, including landscape metrics such as forest patchiness, clumpiness, edge density, and core forest area, can impact bat populations through access to forest habitat, availability of edge environments, and connectivity between roosting and foraging sites (Patriquin and Barclay 2003; Jantzen and Fenton 2013). Together, variation in both habitat availability and landscape configuration provides a mechanistic basis for understanding spatial and temporal differences in bat capture rates independent of disease‐driven dynamics.

To better understand variation in bat capture rates, we considered a suite of ecological and environmental factors that may influence the abundance of species not susceptible to WNS. One potential driver is the decline of WNS‐susceptible species, which may alter community dynamics and resource availability. If reductions in susceptible species decrease competition for prey or roosting space, we predict that non‐susceptible species would increase in capture rates. In addition to biotic interactions, forest availability, composition, and configuration could also impact bat capture rates. We therefore expected that capture rates of non‐susceptible species would be associated with forest characteristics such as percent forest cover, increased forest core area, decreased edge, or decreased clumpiness.

Because these processes can operate differently across spatial scales, we evaluated patterns at both the installation and site levels. Local‐scale dynamics may reflect fine‐scale habitat conditions and species interactions that influence immediate foraging opportunities, whereas broader landscape patterns may reflect variation in habitat availability and configuration across the installation. By examining long‐term capture data across these multiple spatial scales, we aimed to assess how variation in community composition, forest structure, and environmental conditions collectively influence capture rates. This approach provides a more comprehensive understanding of the factors shaping bat communities in a post‐WNS system and offers insight into how species respond to interacting ecological pressures.

## 2 Methods

### 2.1 Study Area

**FIGURE 1:**
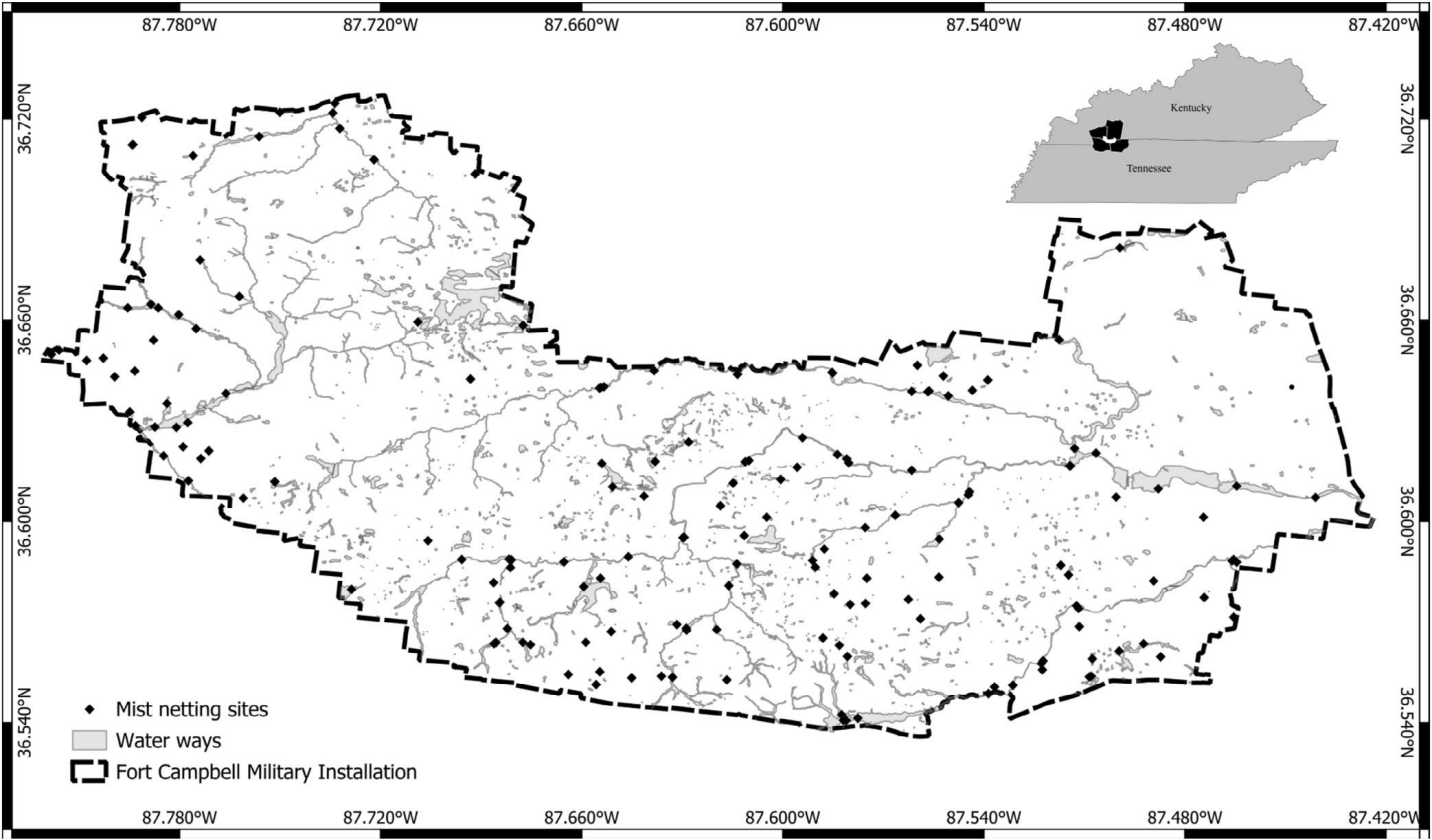
Study area of Fort Campbell Military Installation on the Kentucky‐Tennessee border, highlighting Trigg and Christian Counties in Kentucky, and Stewart and Montgomery Counties in Tennessee.

Our study area was Fort Campbell Military Installation, located on the border of Kentucky and Tennessee, in Trigg and Christian counties in Kentucky and Stewart and Montgomery counties in Tennessee (Figure 1). The installation covers 42,500 ha, with approximately 25,500 ha of military training areas, 10,900 ha of impact zones and ranges, and 6000 ha of urban development (Fort Campbell Fish and Wildlife 2020). Water availability on the installation includes 729 km of streams within nine sub‐watersheds of the Cumberland River and four man‐made lakes totaling about 47 ha. About 20,800 ha are occupied by woodlands, primarily deciduous trees, with pine plantations occupying the southwest region. Grasslands make up approximately 5260 ha, with varying levels of wood succession. Approximately 2400 ha comprise agricultural land and support hay, corn, grain sorghum, and soybean production.

### 2.2 Focal Species

There are currently three endangered bat species that have been captured on Fort Campbell: *M. grisescens* , *M. sodalis* , and *M. septentrionalis* , with two others that have been petitioned for federal protection: *P. subflavus* and *M. lucifugus* (U.S. Fish and Wildlife). Other bat species that have been historically captured on Fort Campbell include *E. fuscus* , *L. noctivagans* , *L. cinereus* , the southeastern bat ( *Myotis austroriparius* ), the Seminole bat ( *Lasiurus seminolus* ), *L. borealis* , and *N. humeralis* . Previous research shows that our focal bat species have overlapping niche spaces while maintaining distinct habitat requirements (Table 1). *E. fuscus* roost in a variety of habitats in winter including caves, mines, tunnels (Beer and Richards 1956; Phillips 1966; Rysgaard 1942; Willis et al. 2006), as well as in buildings (Whitaker and Gummer 1992), tree cavities (Agosta 2002), and rock crevices during the summer (Lausen and Barclay 2002; Neubaum et al. 2006; Valdez and O'Shea 2014). *E. fuscus* are one of the larger‐bodied bat species on the installation and forage in open areas where there is minimal clutter, such as above tree canopies, over open fields, over water, and along forest edges (Duchamp et al. 2004; Farney and Fleharty 1969; Menzel et al. 2005; Norberg and Rayner 1987). Like *E. fuscus* , *M. septentrionalis* utilize caves and mines as winter roosts (Caceres and Barclay 2000; Whitaker Jr and Rissler 1992) and shift to trees in the summer, roosting under loose bark and tree cavities of dead or dying trees (Garcia et al. 2023; Jung et al. 2004; Krynak 2010; Lacki and Schwierjohann 2001). They typically forage in dense, cluttered forests and edge habitats near water (Broders et al. 2006). *P. subflavus* is another species that roosts in caves, culverts, and mines during the winter (Briggler and Prather 2003; Broders et al. 2001; Quinn and Broders 2007) and shifts to a diverse range of roosting sites in the summer, including live and dead foliage, moss, rock crevices, buildings, bridges, and culverts (Ferrara and Leberg 2005; Jones and Pagels 1968; Lacki and Hutchinson 1999; Meierhofer, Johnson, et al. 2019; Meierhofer, Leivers, et al. 2019; Menzel et al. 1999; Veilleux et al. 2003). *P. subflavus* foraging habitat includes open fields, edge habitats, and areas with minimal clutter, often over water (Cable and Willcox 2024; Helms 2010; Rosales 2024; U.S. Fish and Wildlife 2013). *M. sodalis* also rely on caves and abandoned mines in the winter (Brack Jr 1999; Thomson 1982) before transitioning to roosting in snags and underneath the exfoliating bark of dead trees with little to no canopy cover in the summer (Timpone et al. 2010). Their foraging activity is concentrated in core and edge forest habitats and over water (Bergeson et al. 2013; Kniowski and Gehrt 2014; Whitaker Jr and Ritzi 1999; Womack et al. 2013). Similarly, *M. lucifugus* roost in caves and mines during winter (Davis and Hitchcock 1965) and primarily occupy cavities and crevices in dead trees during summer, though they also roost under exfoliating bark less frequently (Bergeson et al. 2013; Broders and Forbes 2004; Crampton and Barclay 1998; Psyllakis and Brigham 2006). They forage low over bodies of water, in edge habitats, and within cluttered forests (Barclay 1991; Brack Jr 1985; Clare et al. 2011; Crampton and Barclay 1998; Kalcounis and Brigham 1995). *L. borealis* roost year‐round in tree foliage, shrubs, and leaf litter (Mills et al. 2013; Shump Jr and Shump 1982). Similarly, *N. humeralis* roost in tree foliage, under exfoliating bark, and within tree cavities and crevices, including snags and leaf litter at the base of trees (Miles et al. 2006; Munzer 2008; Perry and Thill 2008). Both species mainly forage in forested habitats but can also be found foraging in edge habitats, open fields, and over water (Hutchinson and Lacki 1999; Menzel et al. 2005; Walters et al. 2007). Of the focal species in this study, *M. grisescens* is the only species that roosts in caves year‐round (Whitaker et al. 2001). They primarily forage over water, hunting low over aquatic habitats and nearby terrestrial areas (Best et al. 1997; Brack and LaVal 2006; Lacki et al. 1995).

**TABLE 1:** Niche breakdown of bat species included in the study.

Species	Diet	Foraging habitat	Roost habitat	WNS status	Sources
Big brown bat ( *Eptesicus fuscus* )	Coleoptera, Hemiptera, Diptera, Hymenoptera, Homoptera, Lepidoptera	ED, OF, OW	Caves, mines, tunnels, buildings, tree cavities, rock crevices	Low	Agosta (2002), Agosta and Morton (2003), Beer and Richards (1956), Duchamp et al. (2004), Farney and Fleharty (1969), Feldhamer et al. (2009), Lausen and Barclay (2002), Menzel et al. (2005), Norberg and Rayner (1987), Neubaum et al. (2006), Phillips (1966), Rysgaard (1942), Valdez and O'Shea (2014), Whitaker and Gummer (1992), Whitaker (1995), Whitaker and Barnard (2005), Willis et al. (2006)
Eastern red bat ( *Lasiurus borealis* )	Homoptera, Coleoptera, Hymenoptera, Diptera, Lepidoptera	CL, ED, OF, OW	Tree foliage, shrubs, leaf litter	Not affected	Balcombe and Fenton (1988), Carter et al. (2004), Feldhamer et al. (2009), Hutchinson and Lacki (1999), Mager and Nelson (2001), Menzel et al. (2005), Mills et al. (2013), Norberg and Rayner (1987), Shump Jr and Shump (1982), Walters et al. (2007)
Evening bat ( *Nycticeius humeralis* )	Coleoptera, Homoptera, Hemiptera, Tricoptera, Neuropteran	CL, ED, OW	Tree foliage, under bark, cavities and crevices of live, dead or dying trees, snags and leaf litter	Not affected	Boyles and Robbins (2006), Feldhamer et al. (2009), Hein et al. (2009), Menzel et al. (1999), Menzel et al. (2001), Menzel et al. (2005), Miles et al. (2006), Munzer (2008), Norberg and Rayner (1987), Perry and Thill (2008)
Indiana bat ( *Myotis sodalis* )	Diptera, Hymenoptera, Lepidoptera, Coleoptera, Tricoptera, Homoptera, Hemiptera	CL, ED, OW	Caves, mines, cracks and crevices of dead or mostly dead trees, under bark	High	Bergeson et al. (2013), Brack Jr (1999), Feldhamer et al. (2009), Foster and Kurta (1999), Jachowski et al. (2014), Kniowski and Gehrt (2014), Norberg and Rayner (1987), Timpone et al. (2010), Thomson (1982), Vories and USA (1999), Whitaker Jr and Ritzi (1999), Womack et al. (2013)
Gray bat ( *Myotis grisescens* )	Coleoptera, Diptera, Lepidoptera, Tricoptera, Hemiptera, Homoptera, Ephemeroptera, Hymenoptera, Aranaea, Neuroptera, Orthoptera	OW	Caves	Low	Best et al. (1997), Brack and LaVal (2006), Lacki et al. (1995), Norberg and Rayner (1987), Whitaker et al. (2001)
Little brown bat ( *Myotis lucifugus* )	Tricoptera, Diptera, Isoptera, Coleoptera, Ephemeroptera, Lepidoptera, Homoptera	CL, ED, OF, OW	Caves, buildings, cavities/crevices of trees, under exfoliating bark	High	Balcombe and Fenton (1988), Barclay (1991), Bergeson et al. (2013), Brack Jr (1985), Broders and Forbes (2004), Carter et al. (2004), Clare et al. (2011), Crampton and Barclay (1998), Davis and Hitchcock (1965), Feldhamer et al. (2009), Kalcounis and Brigham (1995), Nelson and Gillam (2016), Norberg and Rayner (1987), Psyllakis and Brigham (2006)
Northern myotis ( *Myotis septentrionalis* )	Lepidoptera, Coleoptera, Neuropteran, Diptera, Hemiptera, Homoptera, Hymenoptera	CL, ED, OW	Caves, mines, tree hollows, under bark of dead trees	High	Broders et al. (2006), Caceres and Barclay (2000), Carter et al. (2004), Feldhamer et al. (2009), Foster and Kurta (1999), Garcia et al. (2023), Jung et al. (2004), Krynak (2010), Lacki and Schwierjohann (2001), Norberg and Rayner (1987), Whitaker Jr and Rissler (1992)
Tricolored bat ( *Perimyotis subflavus* )	Coleoptera, Homoptera, Diptera, Hymenoptera, Lepidoptera	ED, OF, OW	Caves, bridges, tree cavities, tree foliage, under tree bark	High	Briggler and Prather (2003), Broders et al. (2001), Cable and Willcox (2024), Carter et al. (2004), Feldhamer et al. (2009), Ferrara and Leberg (2005), Hammesfahr et al. (2022), Helms (2010), Jones and Pagels (1968), Lacki and Hutchinson (1999), Lutsch (2019), Meierhofer, Johnson, et al. (2019), Meierhofer, Leivers, et al. (2019), Menzel et al. (1999), Menzel et al. (2005), Newman (2020), Newman et al. (2021), Norberg and Rayner (1987), Quinn and Broders (2007), Rosales (2024), Sandel et al. (2001), U.S. Fish and Wildlife (2013), Veilleux et al. (2003)

*Note:* Diet is in order of most consumed to least consumed insect order, based on previous studies. Foraging habitats include cluttered forest (CL), edge habitats (ED), open fields (OF), and over water (OW). White‐nose syndrome (WNS) status defined by measured loads of *Pseudogymnoascus destructans*.

We classified each focal species into two susceptibility groups: susceptible (*M. lucifugus, M. septentrionalis, M. sodalis*, and *P. subflavus* ; Bernard et al. 2017; Cheng et al. 2021) and non‐susceptible ( *E. fuscus* , *L. borealis* , *N. humeralis* , and *M. grisescens* ). Due to *M. lucifugus, M. septentrionalis, M. sodalis*, and *P. subflavus* being cave‐dwelling bats during the winter, they are exposed to *Pd* and are some of the hardest hit species, with over 90% population declines in some areas (Francl et al. 2012; Hoyt et al. 2021). While *E. fuscus* and *M. grisescens* are exposed to *Pd*, they have not experienced the drastic population declines that other species have experienced (Bernard et al. 2017; Cheng et al. 2021; Frank et al. 2014; Frick et al. 2017; Hoyt et al. 2021). Therefore, we classified them as non‐susceptible for the purposes of this study. Finally, *L. borealis* and *N. humeralis* are exclusively tree‐roosting species during the winter and are rarely exposed to the fungus (Boyles and Robbins 2006; Mormann and Robbins 2007), and thus were also classified as non‐susceptible.

### 2.3 Capture Data

Capture data was collected by Fort Campbell Fish and Wildlife and Austin Peay State University from 1998 to 2023, with WNS first appearing on the installation in 2013 (with no surveys for years 2007, 2008, 2010, and 2012; 11 years of data both pre‐ and post‐WNS invasion). Capture nights occurred during summer months, with sampling occurring as early as April and as late as September across years. Capture at a given location typically occurred over two consecutive nights, with a maximum of four nights depending on study objectives. Sites were selected based on accessibility (i.e., no roadblock), availability (i.e., no military exercises in the area), and anticipated capture success. Site selection was also guided by targeted study objectives (i.e., capture of specific species). Following the arrival of WNS, survey efforts greatly increased, with the number of sites expanding from 59 pre‐WNS to 191 post‐WNS, resulting in not all sites being sampled every year. Because sampling locations varied among years and were not randomly selected, inference is limited to patterns in capture rates rather than strict population estimates.

Bats were caught via mist nets of varying size (2.6 m height × 4, 6, 9, 12, and 18 m width) placed on flyways such as streams, firebreaks, and roads. The number of suitable flyways determined the number of nets deployed at each sampling location, ranging from two nets at some sites to eight nets at others. Nets were opened 30 min after sunset and monitored for at least 4 h per night. Once captured, each bat was identified to species, and standard morphometric measurements were made. All bats were handled in compliance with the Austin Peay State University Institutional Animal Care and Use Committee (IACUC 20.003R) and followed the Association of Mammalogists Care and Use guidelines (Sikes and The Animal Care and Use Committee of the American Society of Mammalogists 2016). We worked under a Tennessee Wildlife Resources Agency‐issued state scientific collection permit (#2314), Kentucky Department of Fish and Wildlife Resources‐issued state educational wildlife collection permits (#SC2011006, #SC2111014), and federally‐issued United States Fish and Wildlife Service Endangered Species permits (#TE80381A‐2, #ES62026D‐3).

### 2.4 Landscape Variables

We used the National Land Cover Dataset (NLCD, 2001–2021; Dewitz 2023) to calculate selected landscape metrics to quantify forest composition and configuration. The NLCD database provides land cover and land cover change data for the United States at a 30 m × 30 m spatial resolution and is updated every 5 years via Landsat imagery (Xian et al. 2009). We manipulated the NLCD dataset for each available year (2001, 2004, 2006, 2008, 2011, 2013, 2016, 2019, and 2021) by reclassifying pixels to forest (deciduous, evergreen, mixed), developed (low, medium, high intensity and open developed space), grassland (shrub/scrub, grassland/herbaceous), agriculture (pasture/hay, cultivated crops), water (open water), and other (perennial ice/snow, barren land, woody wetlands, emergent herbaceous wetlands) using the *raster* package in R v4.4.1 (Hijams 2024). As most of our focal bat species reside in deciduous forests, we also created an additional reclassified layer of deciduous forest with all other classifications set as “other.”

Given the variation in foraging and roosting strategies among our focal species, we chose different landscape metrics at the installation (annual capture rate across the entire installation area) and site (annual capture rate per sampling site) levels. Metrics were selected to capture the variability in forest composition and configuration that may influence capture rates at different spatial scales among the focal species. We determined metrics relevant at the installation level included the proportion of forest (Ethier and Fahrig 2011; Patriquin and Barclay 2003), the percent of each patch of forest that was represented by core habitat (defined as pixels ≥ 30 m from the patch edge, corresponding to one pixel based on NLCD resolution), forest patchiness, forest patch clumpiness, and largest forest patch index (Crome and Richards 1988; De Jong 1995; Gorresen and Willig 2004; Hogberg et al. 2002); these metrics were calculated for both the total forest classification and the deciduous forest only classification. At the site level, landscape metrics included the proportion of forest, percentage of core forest, forest edge density, and largest forest patch index of both total forest and deciduous forest within a 1000 m buffer of each sampling site (Aldridge and Rautenbach 1987; Rodríguez‐San Pedro and Simonetti 2015).

We used the *landscapemetrics* package (Hesselbarth et al. 2019) in R to calculate the proportion of total forest (classified as deciduous, evergreen, or mixed) and the proportion of only deciduous forest for the entire installation and within a 1000 m buffer surrounding each sampling site for each capture year. We used the *sf* package (Pebesma and Bivand 2003) in R to create a 1000 m buffer around each sampling site; a 1000 m buffer was chosen based on in‐house telemetry data of *P. subflavus* movement distances from capture location to roost trees gathered in 2020 and 2021 (Williams et al. 2025). Land cover classification was based on NLCD data from 2001, 2004, 2006, 2008, 2011, 2016, 2019, and 2021, with each capture year being assigned to the closest available NLCD year. We then calculated total forest patchiness (function “lsm_c_pd”), forest patch clumpiness (“lsm_c_clumpy”), largest forest patch index (“lsm_c_lpi”), and core percent (“lsm_c_cpland”) at the installation level, and edge density (“lsm_c_ed”), core forest area (“lsm_c_area_mn”), and largest forest patch index (“lsm_c_lpi”) at the site level for each capture year using 8‐direction connectivity.

### 2.5 Environmental Data

Temperature extremes and precipitation influence the timing and intensity of bat foraging activity (Frick et al. 2012; Grindal et al. 1992). To account for potential variation in annual capture rates driven by local weather, we gathered nightly temperature (°C) and daily precipitation (cm) data during summer months (April–September) from the National Oceanic and Atmospheric Administration (weather station ID 74671013806; resolution: hourly). To represent biologically meaningful annual summaries, we determined the maximum nightly temperature value for each day of the summer netting season and then calculated the mean maximum daily temperature for each year. We also calculated the mean of the total daily precipitation for the summer netting season for each year.

### 2.6 Statistical Analyses

To test our hypotheses, we first calculated annual capture rates for each species at the installation level by dividing the number of individuals of each species captured on Fort Campbell each year by the total number of nights netted × total nets deployed that year (referenced as “net nights” throughout) on the installation that year. At the site level, annual capture rate was calculated by dividing the number of individuals of each species captured at each sampling site each year by the number of net nights at each site each year (total number of nights netted that year at that site × number of nets deployed that year at that site). We used capture rate rather than raw abundance because, like everywhere else in North America, Fort Campbell increased survey efforts after the arrival of WNS to the state. This increased effort occurred via the use of more nets per site when able, an increase in total annual net nights, and an increase in the number of sites sampled across the installation. We developed a suite of generalized linear models to evaluate factors influencing capture rates for each of our focal species (*E. fuscus, M. grisescens, L. borealis*, *N. humeralis* ) at two spatial scales: the installation level and site level. At the installation level, the response variable was the annual capture rate for each species across the entire installation, and the sampling unit was year. At the site level, the response variable was the annual capture rate (or abundance) at individual mist‐netting locations, and the sampling unit was site‐year. For each focal species and spatial scale, we constructed a suite of models that included a single focal predictor per model to evaluate the specific ecological drivers (Table 2). Focal predictors included either (1) annual capture rates of WNS‐susceptible species ( *M. lucifugus* , *M. septentrionalis* , *M. sodalis* , *P. subflavus* , and all susceptible species combined) or (2) landscape metrics describing forest composition and configuration. All models also included mean daily maximum air temperature (°C) and total daily precipitation (mm) as covariates. Prior to modeling, we assessed multicollinearity among predictors using correlation analysis and found no strong correlations (all |*r* ^2^| < 0.60).

**TABLE 2:** Variables used to determine influence on annual capture rates of four bat species ( *Eptesicus fuscus* , *Myotis grisescens* , *Lasiurus borealis* , *Nycticeius humeralis* ) during April to September 1998–2023 on Fort Campbell Military Installation at the installation and site (within 1000 m of mist‐netting site) spatial scales.

Variable	Covariate description	Installation level covariate range	Site level covariate range	Source	R function
Capture rate of all susceptible bat species	Capture rate of all susceptible species	0.00–0.17	0.05–5.00	This study	—
Capture rate of *Myotis lucifugus*	Capture rate of *M. lucifugus*	0.00–0.01	0.00–0.75	This study	—
Capture rate of *Myotis septentrionalis*	Capture rate of *M. septentrionalis*	0.00–0.05	0.00–1.00	This study	—
Capture rate of *Myotis sodalis*	Capture rate of *M. sodalis*	0.00–0.01	0.00–0.25	This study	—
Capture rate of *Perimyotis subflavus*	Capture rate of *P. subflavus*	0.00–0.12	0.00–5.75	This study	—
Pre‐ or post‐white‐nose syndrome	Binary variable if capture year is pre‐ or post‐white‐nose syndrome invasion	0 or 1	0 or 1	If capture year is before/after 2013	—
Maximum temperature	Mean of daily maximum temperatures (°C) during capture months	19.75–22.82	19.75–22.82	NOAA	—
Total daily precipitation	Mean total daily precipitation (cm) during capture months	79.50–150.22	79.50–150.22	NOAA	—
Proportion of total forest	Proportion of study area that is total forest for that capture year	0.15–0.16	0.00–0.57	NLCD	—
Proportion of deciduous forest	Proportion of the study area that is deciduous forest that capture year	0.53–0.55	0.05–0.93	NLCD	—
Total forest patchiness	# patches of total forest per 100 ha in the study area that capture year	3.02–3.33	—	NLCD	lsm_c_pd
Deciduous forest patchiness	# patches of deciduous forest per 100 ha in the study area that capture year	3.88–4.08	—	NLCD	lsm_c_pd
Total forest patch clumpiness	Clumpiness of total forest in the study area that capture year	0.80–0.81	—	NLCD	lsm_c_clumpy
Deciduous forest patch clumpiness	Clumpiness of deciduous forest in the study area that capture year (−1 disaggregation, 0 randomly distributed, 1 aggregation)	0.77–0.78	—	NOAA	lsm_c_clumpy
Largest patch index of total forest	How much of the study area the largest patch of total forest occupies that capture year (ranges from 0 to 100)	10.68–11.89	3.05–99.72	NLCD	lsm_c_lpi
Largest patch index of deciduous forest	How much of the study area the largest patch of total forest occupies that capture year (ranges from 0 to 100)	6.54–7.29	2.94–88.28	NOAA	lsm_c_lpi
Percent of core total forest	A percentage (%) of the study area is core total forest that capture year	39.18–41.12	—	NLCD	lsm_c_cpland
Percent of core deciduous forest	A percentage (%) of the study area is core deciduous forest that capture year	27.87–28.80	—	NLCD	lsm_c_cpland
Core total forest	Core total forest habitat within study area that capture year (ha)	—	1.56–323.46	NLCD	lsm_c_area_mn
Core deciduous forest	Core deciduous forest habitat within study area that capture year (ha)	—	1.11–281.52	NLCD	lsm_c_area_mn
Edge total forest density	Edge density (m ha^−1^) of total forest within study area that capture year	—	1.94–135.17	NLCD	lsm_c_ed
Edge deciduous forest density	Edge density (m ha^−1^) of deciduous forest within study area that capture year	—	28.98–135.44	NLCD	lsm_c_ed

*Note:* Data were calculated in this study, from the National Oceanic and Atmospheric Administration (NOAA), or National Land Cover Dataset (2001–2021; NLCD).

At the installation level, we fit 16 candidate models for each focal species (Tables S1–S4). These included five models incorporating susceptible species capture rates (each with an interaction term of a binary pre/post‐WNS variable), ten models including landscape metrics (proportion of total or deciduous forest, patchiness of total or deciduous forest, clumpiness of total or deciduous forest, total % core of total or deciduous forest, and largest patch index of total or deciduous forest), and one baseline model including pre/post‐WNS, temperature, and precipitation. Installation‐level models were fit using a Gaussian distribution with a log link to account for non‐normality in the response variable. All continuous predictors were centered and scaled.

At the site level, we fit an equivalent set of 16 models for each focal species (Tables S6–S10) using a zero‐inflated negative binomial framework (family = “nbinom1”) to account for overdispersion and excess zeros in the data. Models were implemented using the glmmTMB package in R (Brooks et al. 2017). The conditional (count) component included temperature, precipitation, and the focal predictor, while the zero‐inflation component included only the focal predictor. We included log‐transformed annual net‐nights as an offset to account for variation in sampling effort among sites and years. All continuous predictors were centered and scaled prior to analysis.

Model selection was based on second‐order Akaike's Information Criterion corrected for small sample sizes (AICc; Akaike 1973). For each species and spatial scale, the model with the lowest AICc value was considered the top‐supported model. When multiple models were within ΔAICc ≤ 2, we used AICc weights to evaluate relative support and identified the model with the highest weight as the most supported (Burnham and Anderson 2002). We interpreted parameter estimates, effect directions, and statistical significance within top‐supported models considering statistical significance at α = 0.05. All analyses were conducted in R version 4.4.1 (R Core Team 2024).

## 3 Results

**FIGURE 2:**
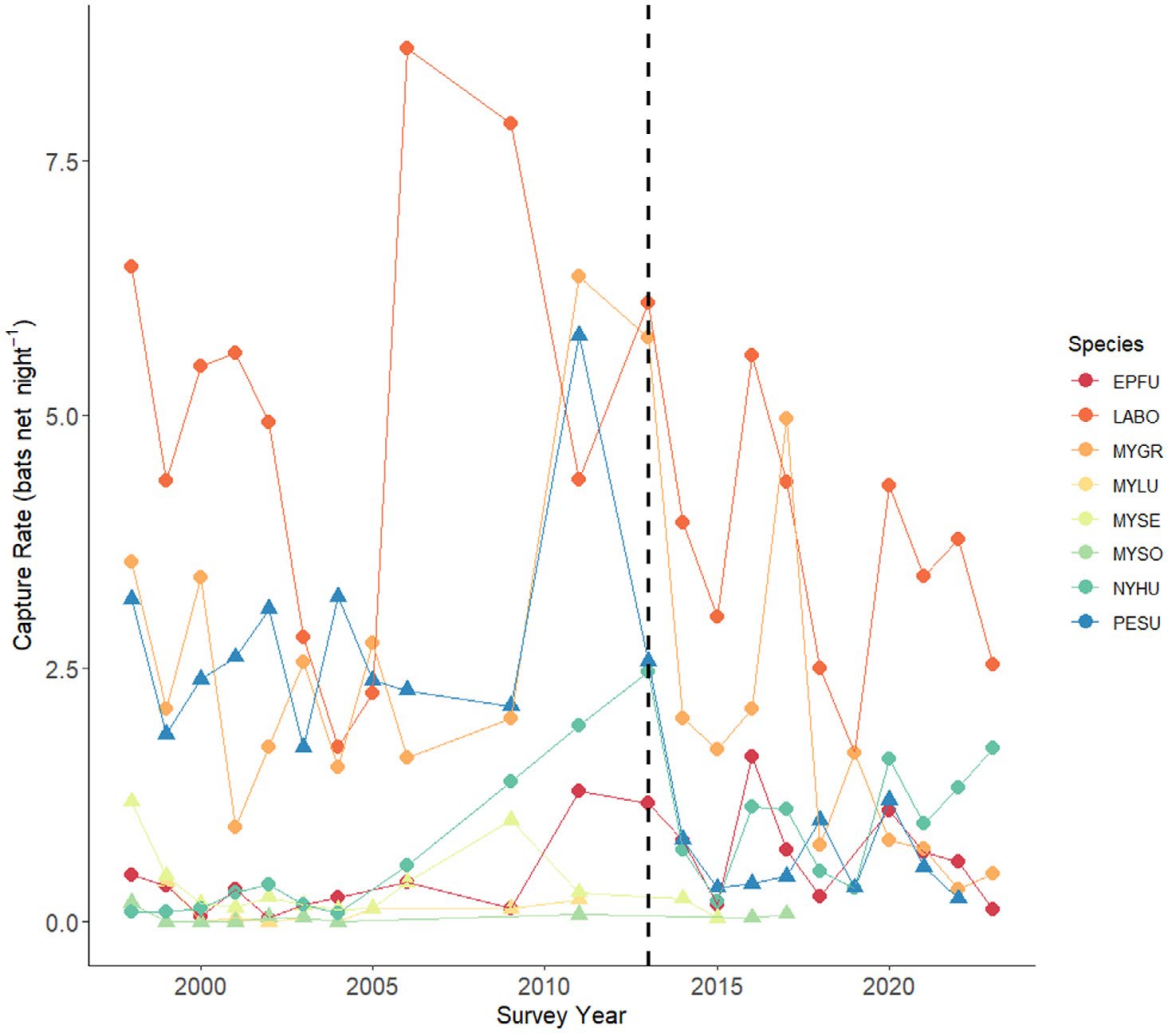
Capture rates (total captures/# of net nights each year) of four non‐susceptible bat species to white‐nose syndrome (EPFU: *Eptesicus fuscus* , MYGR: *Myotis grisescens* , LABO: *Lasiurus borealis* , NYHU: *Nycticeius humeralis* ) and four susceptible bat species (MYLU: *Myotis lucifugus* , MYSE: *Myotis septentrionalis*, MYLU: *Myotis sodalis* , PESU: *Perimyotis subflavus* ) during April to September 1998–2023 on Fort Campbell Military Installation, Kentucky. Susceptible species are indicated by triangles, non‐susceptible species are indicated by circles, and the vertical dotted line indicates white‐nose syndrome invasion on Fort Campbell (2013).

There was an increase in capture effort following the onset of WNS, with both the number of nights and number of nets increasing with the onset of WNS on Fort Campbell (Figure S1). Over the 11 years of data collection prior to 2013, an average of 13 sites were visited annually (minimum = 1, maximum = 24, total = 180) with 2–12 nets per site (17,830 net nights total). Over the 11 years of data collection post‐2013, however, an average of 22 sites were visited annually (minimum = 2, maximum = 54, total = 262), with 1–18 nets per site (61,021 net nights total). There was a mean minimum distance of 536.6 m between sites (range: 11.1–2737.4 m). Despite the heightened effort, the total number of individual bats captured showed only a slight increase, from 2237 bats over 11 years pre‐WNS to 2419 bats over 11 years post‐WNS (Table 3). However, overall captures of susceptible species declined sharply by 69.27% (pre‐WNS: 641 individuals over 11 years; post‐WNS: 197 individuals). In contrast, non‐susceptible species experienced a 39.22% increase in captures (pre‐WNS: 1614 individuals over 11 years; post‐WNS: 2244 individuals), highlighting a notable shift in species composition following WNS invasion at Fort Campbell (Figure 2).

**TABLE 3:** Total captures (*N*) and mean capture rates (total captures/# of netting nights × number of nets) with standard deviation (SD) of four non‐susceptible bat species ( *Eptesicus fuscus* , *Myotis grisescens* , *Lasiurus borealis* , *Nycticeius humeralis* ) and four susceptible bat species ( *Myotis lucifugus* , *Myotis septentrionalis* , *Myotis sodalis* , *Perimyotis subflavus* ) to white‐nose syndrome during April to September 1998–2023 on Fort Campbell Military Installation, Kentucky.

Species	Pre‐WNS			Post‐WNS		
	*N*	Mean capture rate	SD	*N*	Mean capture rate	SD
Non‐susceptible						
*Eptesicus fuscus*	59	0.005	0.006	221	0.007	0.009
*Lasiurus borealis*	974	0.082	0.066	1111	0.078	0.119
*Myotis grisescens*	486	0.046	0.043	587	0.052	0.122
*Nycticeius humeralis*	77	0.006	0.007	303	0.020	0.025
Susceptible						
*Myotis lucifugus*	15	0.002	0.002	0	0.000	0.000
*Myotis septentrionalis*	63	0.008	0.013	8	0.001	0.001
*Myotis sodalis*	5	0.001	0.002	4	0.001	0.001
*Perimyotis subflavus*	558	0.046	0.034	185	0.020	0.028
Total	2237	0.026	0.042	2419	0.034	0.078

### 3.1 Installation Level

At the installation level, drivers of capture rates differed among focal species. Annual capture rates of *M. grisescens* (Table S1) and *L. borealis* (Table S2) were strongly associated with capture rates of susceptible species, while *N. humeralis* (Table S3) and *E. fuscus* (Table S4) were more strongly associated with landscape characteristics. However, the positive relationships observed between susceptible and non‐susceptible species do not support ecological release, which would predict negative associations.

**FIGURE 3:**
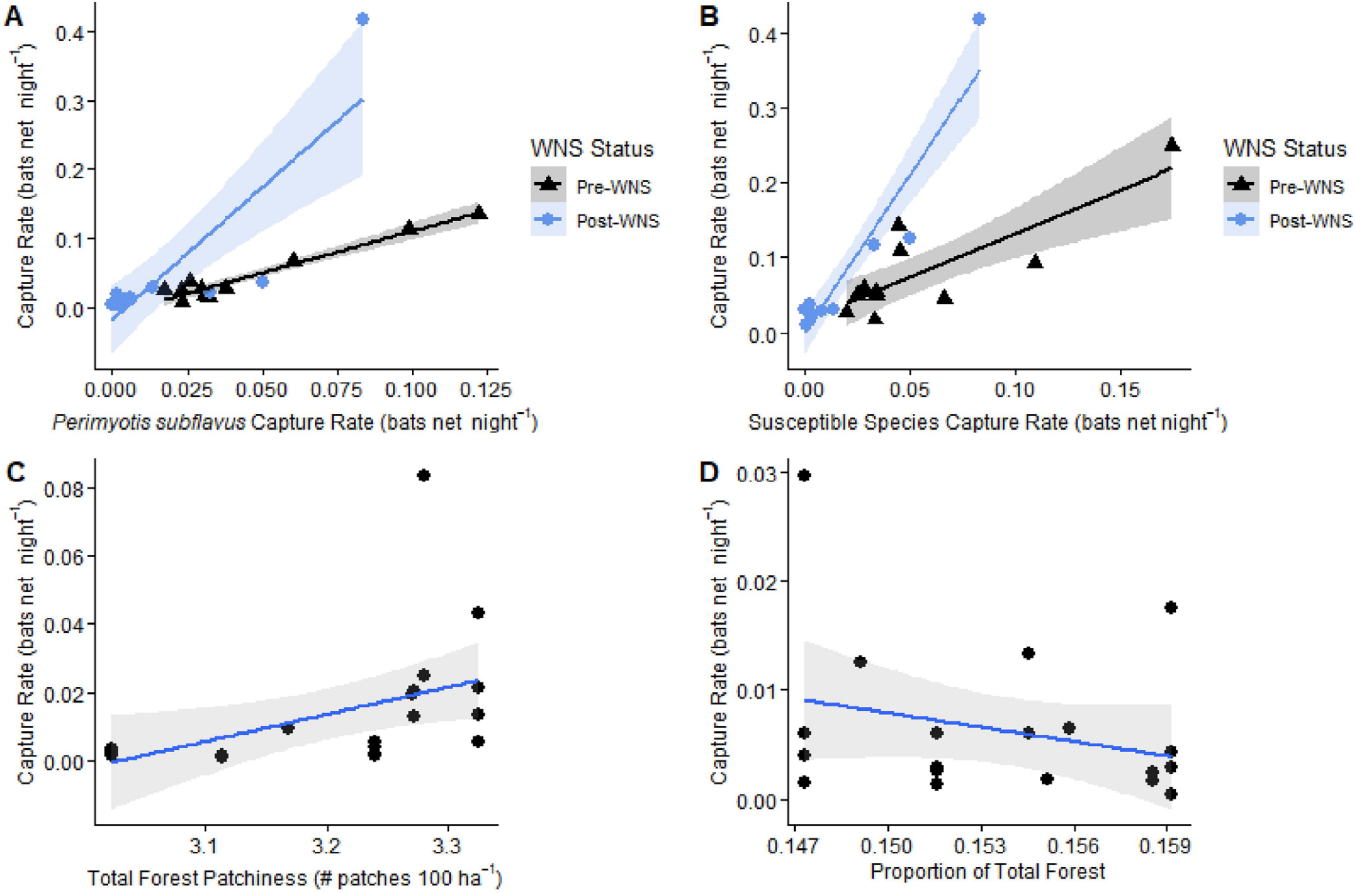
Relationship between annual summer (April to September) capture rates (# captures/# net nights) of: (A) *Myotis grisescens* and *Perimyotis subflavus* capture rates, (B) *Lasiurus borealis* and total white‐nose syndrome susceptible species capture rates, (C) *Nycticeius humeralis* and total forest patchiness (number of patches per 100 ha), and (D) *Eptesicus fuscus* and proportion of total forest at the installation level spatial scale on Fort Campbell Military Installation, Kentucky from 1998 to 2023, with white‐nose syndrome first observed in 2013. Shaded areas represent 95% confidence intervals.

Specifically, annual capture rates of *M. grisescens* increased with capture rates of *P. subflavus* (β = 22.40 ± 5.22 SE, *p* < 0.001; Table S5; Figure 3A), with a stronger positive relationship following WNS occurrence (baseline factor: pre‐WNS; β = 41.65 ± 8.54 SE, *p* < 0.001). Despite this interaction, overall capture rates of *M. grisescens* declined post‐WNS (baseline factor: pre‐WNS; β = −1.88 ± 0.58 SE, *p* = 0.005). There was no influence of mean daily maximum air temperature (*p* = 0.258) or total daily precipitation (*p* = 0.760) on capture rates. Similarly, capture rates of *L. borealis* increased with capture rates of all susceptible species (β = 9.21 ± 3.20 SE, *p* = 0.011; Table S5; Figure 3B), with a stronger increase post‐WNS (baseline factor: pre‐WNS; β = 23.52 ± 5.26 SE, *p* < 0.001). There was no independent effect of WNS status (*p* = 0.220), air temperature (*p* = 0.696), or precipitation on capture rates (*p* = 0.992). Capture rates of *N. humeralis* were best explained by landscape composition, increasing with forest patchiness (β = 4.56 ± 0.64 SE, *p* < 0.001; Table S5; Figure 3C) and precipitation (β = 1.71 ± 0.18 SE, *p* < 0.001); there was no effect however of air temperature (*p* = 0.347). Finally, capture rates of *E. fuscus* decreased with increasing proportion of total forest cover on the installation (β = −3.53 ± 1.51 SE, *p* = 0.033; Table S5; Figure 3D), and were not significantly influenced by air temperature (*p* = 0.126) or precipitation (*p* = 0.119).

### 3.2 Site Level

At the site level, patterns were broadly consistent with those observed at the installation scale, with species responses differing between susceptible species capture rates and landscape composition. Capture rates of *M. grisescens* were most strongly associated with capture rates of *P. subflavus* (Table S6), while *L. borealis, N. humeralis*, and *E. fuscus* were primarily associated with local habitat characteristics, particularly the proportion of total forest within a 100 m buffer (Tables S7–S9).

**FIGURE 4:**
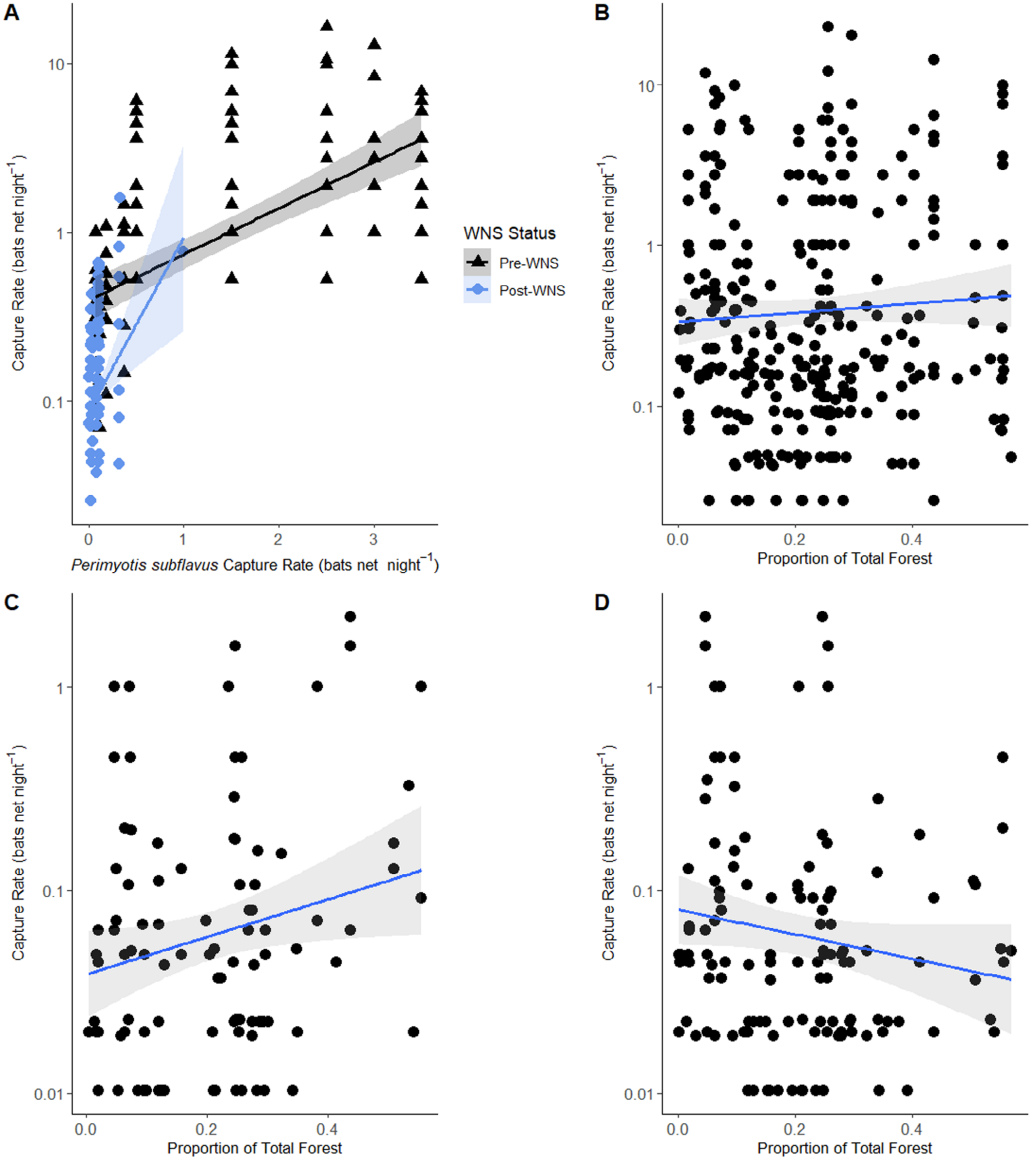
Relationship between annual summer (April to September) capture rates (log(# captures/# net nights)) of (A) *Myotis grisescens* with capture rates of *Perimyotis subflavus* , (B) *Lasiurus borealis* (C) *Eptesicus fuscus* , and (D) *Nycticeius humeralis* and proportion of total forest (deciduous, evergreen, and mixed) within 1000 m buffers at the site level spatial scale on Fort Campbell Military Installation, Kentucky from 1998 to 2023, with white‐nose syndrome first observed in 2013. Shaded areas represent 95% confidence intervals. Capture rate data of focal species are log‐transformed given the data are zero‐inflated.

Capture rates of *M. grisescens* were positively associated with *P. subflavus* (β = 1.21 ± 0.11 SE, *p* < 0.001; Table S10; Figure 4A), with this relationship becoming stronger following WNS invasion (interaction term: β = 14.01 ± 2.33 SE, *p* < 0.001). Despite this, overall capture rates of *M. grisescens* declined post‐WNS (β = −1.86 ± 0.12 SE, *p* < 0.001). Capture rates also decreased with increasing mean daily maximum air temperature (β = −0.19 ± 0.07 SE, *p* = 0.005), while precipitation had no significant effect (*p* = 0.070). None of the predictors significantly influenced presence–absence for this species (all *p* > 0.05). Capture rates of *L. borealis* and *E. fuscus* increased with the proportion of forest surrounding each site ( *L. borealis* : β = 0.11 ± 0.06 SE, *p* = 0.044; Table S10; Figure 4B; *E. fuscus* : β = 0.32 ± 0.10 SE, *p* = 0.001; Table S10; Figure 4C). Temperature did not significantly influence capture rates for either species (both *p* > 0.05). Precipitation had no effect on *E. fuscus* (*p* = 0.083) but was negatively associated with capture rates of *L. borealis* (β = −0.41 ± 0.06 SE, *p* < 0.001). Finally, capture rates of *N. humeralis* decreased with increasing temperature (β = −0.45 ± 0.08 SE, *p* < 0.001; Table S10; Figure 4D) and increased with precipitation (β = 0.21 ± 0.08 SE, *p* = 0.012), with no significant effect of forest cover (*p* = 0.092). No variables significantly influenced presence–absence for *L. borealis* , *E. fuscus* , or *N. humeralis* (all *p* > 0.05).

## 4 Discussion

The purpose of this study was to investigate how WNS and landscape composition and configuration were affecting capture rates of non‐susceptible bat species on Fort Campbell Military Installation. We evaluated these relationships at both the installation‐wide and site‐specific spatial scales to assess whether declines in WNS‐susceptible species were associated with increases in non‐susceptible species, as predicted under ecological release, or whether landscape composition and configuration better explained variation in capture rates. The results were mixed, with patterns varying by spatial scale and species, and overall suggest that bat community dynamics following a major disease are shaped by environmental conditions and habitat structure rather than by ecological release from competition.

Interestingly, capture rates of two non‐susceptible species increased after WNS occurrence at both the installation and site‐level spatial scales, contrary to our proposed hypothesis of ecological release. Capture rates of *M. grisescens* and *L. borealis* were positively associated with capture rates of susceptible species at the installation level (Figure 3A,B), and *M. grisescens* remained positively associated with *P. subflavus* at the site level (Figure 4A). These results were unexpected, as we had expected a negative correlation, which would be indicative of ecological release from competition (Connell 1961, 1983; Macarthur and Levins 1967) as observed in previous studies in Kentucky (Thalken et al. 2018), New York (Ford et al. 2011; Jachowski et al. 2014), Ontario (Morningstar et al. 2019), and Pennsylvania (Hauer et al. 2019). These positive correlations may reflect shared environmental preferences or prey availability rather than direct competition. Many bat species in the eastern United States rely on forests for roosting and/or foraging (Loeb and O'Keefe 2006; Yates and Muzika 2006), and their niche overlap may contribute to their coexistence (Morris et al. 2008; Novella‐Fernandez et al. 2020), which could be driving the observed patterns in capture rates. When resources are abundant but spatially heterogeneous, species with similar niche requirements may partition forage resources or roosting locations by using different foraging strategies (e.g., gleaning, aerial hawking), roosting at different heights, or being active at slightly different times. In these instances, positive relationships between capture rates may not necessarily indicate potential competition to be released from but rather lack of interspecific exclusion.

In contrast to the limited support for ecological release, landscape composition and configuration explained variation in capture rates for several species across both spatial scales. At the installation level, *N. humeralis* capture rates increased with forest patchiness (Figure 3C), whereas *E. fuscus* capture rates declined with increasing forest cover (Figure 3D). At the site level, capture rates of *L. borealis* and *E. fuscus* increased with the proportion of forest surrounding sampling locations (Figure 4B,C). These patterns are consistent with prior studies linking bat activity to forest structure and composition (Heim et al. 2015; Neece et al. 2018; Treitler et al. 2016), and align with known habitat preferences of these insectivorous species which favor foraging in edge‐rich and cluttered habitats (Cable and Willcox 2024; Helms 2010; Menzel et al. 2005; Table 1). Greater patchiness of forested areas may create a more heterogeneous landscape, increasing the amount of forest edge and connecting corridors. These features likely enhance foraging efficiency for edge‐adapted species by concentrating insect prey along edges, where vegetation structure and microclimates support high insect abundance, and by facilitating maneuverability during flight (Grindal and Brigham 1999). The negative relationship between *E. fuscus* and total forest cover at the installation level aligns with their preference for foraging in edge habitats and above the canopy, where insect abundance tends to be high (Agosta 2002; Duchamp et al. 2004; Farney and Fleharty 1969; Menzel et al. 2005; Norberg and Rayner 1987; Table 1). These results are consistent with previous findings that landscape features, particularly forest habitats, play a central role in shaping bat species' distributions and activity (Fenton and Barclay 1980; LaVal and LaVal 1980).

Several factors may explain these unexpected results with non‐susceptible species' capture rates following a positive correlation with susceptible species capture rates. One possible explanation is the increase in mist‐netting effort after the arrival of WNS to Fort Campbell, which resulted in higher sampling nights per year and more sites surveyed each year. Though we tried to address this increase in sampling effort post‐WNS by standardizing the capture rates, it is possible that the overall increase in capture rates, regardless of species and WNS status, reflected survey intensity rather than population trends. It may also be due to the nonrandom nature in which sites were selected. Over the years, sites were selected to maximize the chance of capturing WNS‐impacted species. Thus, not every site was visited each year. Additionally, the severe population declines of susceptible species likely led to very low capture rates in post‐WNS years, which could have reduced the statistical power of the statistical models. For example, post‐WNS, many of the susceptible species disappeared from many sampling sites, which may have skewed model results toward effects of conspecific relationships before WNS when more data was available. Finally, if capture rates themselves were influenced by environmental conditions which could skew detectability of all bat species, we expect rates to increase or decrease in parallel, regardless of susceptibility levels. However, as we consider the environmental variables of temperature and precipitation in all models, we suspect this is not the case.

While much of our conservation focus in North America has rightly been on supporting species severely impacted by WNS, it is equally critical to not overlook non‐susceptible species, many of which are also declining due to habitat loss and degradation due to land use change. Conserving critical foraging and roosting habitats is a key strategy for slowing or possibly reversing the ongoing global decline in bat populations. These findings highlight the importance of long‐term monitoring using standardized methods to accurately track population trends and distinguish true ecological responses from sampling effects. Integrated conservation planning that balances disease mitigation with habitat protection needs to be at the forefront of bat conservation, and military lands such as Fort Campbell could serve as models for balancing land use with species protection.

## Author Contributions

**Dakota J. Van Parys:** data curation (lead), formal analysis (lead), investigation (equal), writing – original draft (lead). **Sarah C. Williams:** data curation (equal), investigation (equal), writing – review and editing (equal). **Catherine G. Haase:** data curation (equal), formal analysis (equal), investigation (equal), project administration (equal), supervision (lead), writing – review and editing (lead).

## Funding

This work was supported by the U.S. Department of Defense, IGSA‐A60RC‐23‐ENV‐001. Austin Peay State University.

## Conflicts of Interest

The authors declare no conflicts of interest.

## Supporting information


**Table S1:** Suite of models for *Myotis grisescens* relating annual capture rates at the installation level spatial scale to capture rates of susceptible species to white‐nose syndrome (WNS) ( *Myotis lucifugus* , *Myotis septentrionalis* , *Myotis sodalis* , *Perimyotis subflavus* ), total susceptible species capture rates, or various landscape metrics on Fort Campbell Military Installation from 1998 to 2023. All models included mean daily maximum temperature (°C) and total daily precipitation (cm) as covariates. Differences in Akaike information criterion corrected for small sample sizes (ΔAICc), log‐likelihood (LL), number of parameters (K), and AICc weights (w_t_) are reported.
**Table S2:** Suite of models for *Lasiurus borealis* relating annual capture rates at the installation level spatial scale to capture rates of susceptible species to white‐nose syndrome (WNS) ( *Myotis lucifugus* , *Myotis septentrionalis* , *Myotis sodalis* , *Perimyotis subflavus* ), total susceptible species capture rates, or various landscape metrics on Fort Campbell Military Installation from 1998 to 2023. All models included mean daily maximum temperature (°C) and total daily precipitation (cm) as covariates. Differences in Akaike information criterion corrected for small sample sizes (ΔAICc), log‐likelihood (LL), number of parameters (K), and AICc weights (w_t_) are reported.
**Table S3:** Suite of models for *Nycticeius humeralis* relating annual capture rates at the installation level spatial scale to capture rates of susceptible species to white‐nose syndrome (WNS) ( *Myotis lucifugus* , *Myotis septentrionalis* , *Myotis sodalis* , *Perimyotis subflavus* ), total susceptible species capture rates, or various landscape metrics on Fort Campbell Military Installation from 1998 to 2023. All models included mean daily maximum temperature (°C) and total daily precipitation (cm) as covariates. Differences in Akaike information criterion corrected for small sample sizes (ΔAICc), log‐likelihood (LL), number of parameters (K), and AICc weights (w_t_) are reported.
**Table S4:** Suite of models for *Eptesicus fuscus* relating annual capture rates at the installation level spatial scale to capture rates of susceptible species to white‐nose syndrome (WNS) ( *Myotis lucifugus* , *Myotis septentrionalis* , *Myotis sodalis* , *Perimyotis subflavus* ), total susceptible species capture rates, or various landscape metrics on Fort Campbell Military Installation from 1998 to 2023. All models included mean daily maximum temperature (°C) and total daily precipitation (cm) as covariates. Differences in Akaike information criterion corrected for small sample sizes (ΔAICc), log‐likelihood (LL), number of parameters (K), and AICc weights (w_t_) are reported.
**Table S5:** Variable parameter estimates (β), standard errors (SE), and *p*‐values of the top models relating annual capture rates at the installation level spatial scale of four non‐susceptible bat species to susceptible species capture rates or landscape metrics on Fort Campbell Military Installation from 1998 to 2023. Pre or post terms designate capture years before (< 2013) or after (> 2013) WNS invasion. Mean daily maximum air temperature and total daily precipitation were scaled.
**Table S6:** Suite of models for *Myotis grisescens* relating annual capture rates at the site level spatial scale to capture rates of susceptible species to white‐nose syndrome (WNS) ( *Myotis lucifugus* (MYLU), *Myotis septentrionalis* (MYSE), *Myotis sodalis* (MYSO), *Perimyotis subflavus* (PESU)), total susceptible species capture rates, or various landscape metrics on Fort Campbell Military Installation from 1998 to 2023. All models included an interaction of a binary variable of pre‐ or post‐WNS invasion and mean daily maximum temperature (°C) and total daily precipitation (cm) as covariates. Differences in Akaike information criterion corrected for small sample sizes (ΔAICc), log‐likelihood (LL), number of parameters (K), and AICc weights (w_t_) are reported.
**Table S7:** Suite of models for *Lasiurus borealis* relating annual capture rates at the fine site level scale to capture rates of susceptible species to white‐nose syndrome (WNS) ( *Myotis lucifugus* (MYLU), *Myotis septentrionalis* (MYSE), *Myotis sodalis* (MYSO), *Perimyotis subflavus* (PESU)), total susceptible species capture rates, or various landscape metrics on Fort Campbell Military Installation from 1998 to 2023. All models included an interaction of a binary variable of pre‐ or post‐WNS invasion and mean daily maximum temperature (°C) and total daily precipitation (cm) as covariates. Differences in Akaike information criterion corrected for small sample sizes (ΔAICc), log‐likelihood (LL), number of parameters (K), and AICc weights (w_t_) are reported.
**Table S8:** Suite of models for *Nycticeius humeralis* relating annual capture rates at the fine site level scale to capture rates of susceptible species to white‐nose syndrome (WNS) ( *Myotis lucifugus* (MYLU), *Myotis septentrionalis* (MYSE), *Myotis sodalis* (MYSO), *Perimyotis subflavus* (PESU)), total susceptible species capture rates, or various landscape metrics on Fort Campbell Military Installation from 1998 to 2023. All models included an interaction of a binary variable of pre‐ or post‐WNS invasion and mean daily maximum temperature (°C) and total daily precipitation (cm) as covariates. Differences in Akaike information criterion corrected for small sample sizes (ΔAICc), log‐likelihood (LL), number of parameters (K), and AICc weights (w_t_) are reported.
**Table S9:** Suite of models for *Eptesicus fuscus* relating annual capture rates at the fine site level scale to capture rates of susceptible species to white‐nose syndrome (WNS) ( *Myotis lucifugus* (MYLU), *Myotis septentrionalis* (MYSE), *Myotis sodalis* (MYSO), *Perimyotis subflavus* (PESU)), total susceptible species capture rates, or various landscape metrics on Fort Campbell Military Installation from 1998 to 2023. All models included an interaction of a binary variable of pre‐ or post‐WNS invasion and mean daily maximum temperature (°C) and total daily precipitation (cm) as covariates. Differences in Akaike information criterion corrected for small sample sizes (ΔAICc), log‐likelihood (LL), number of parameters (K), and AICc weights (w_t_) are reported.
**Table S10:** Variable parameter estimates (β), standard errors (SE), and *p*‐values of the top models relating annual capture rates at the fine site level scale of four non‐susceptible bat species to white‐nose syndrome (WNS) on Fort Campbell Military Installation from 1998 to 2023. Pre or post terms designate capture years before (< 2013) or after (> 2013) WNS invasion.
**Figure S1:** Total netting nights (sampled nights × number of nets) per year during April–September 1998–2023 on Fort Campbell Military Installation, Kentucky. Vertical dashed line indicates white‐nose syndrome invasion on Fort Campbell (2013).

## Data Availability

Required data is available via data Dryad. https://doi.org/10.5061/dryad.98sf7m0z8.
