# Supplementary material for: Shifts in Southeastern Bat Communities Driven by Landscape Composition Rather Than Ecological Release
Source: Ecol Evol. 2026 Jul 31;16(8):e74129. doi: 10.1002/ece3.74129 (PMC13428179; doi:10.1002/ece3.74129)
Supplement: Supplementary file 1 — Table S1: Suite of models for Myotis grisescens relating annual capture rates at the installation level spatial scale to capture rates of susceptible species to white‐nose syndrome (WNS) ( Myotis lucifugus , Myotis septentrionalis , Myotis sodalis , Perimyotis subflavus ), total susceptible species capture rates, or various landscape metrics on Fort Campbell Military Installation from 1998 to 2023. All models included mean daily maximum temperature (°C) and total daily precipitation (cm) as covariates. Differences in Akaike information criterion corrected for small sample sizes (ΔAICc), log‐likelihood (LL), number of parameters (K), and AICc weights (wt) are reported. Table S2: Suite of models for Lasiurus borealis relating annual capture rates at the installation level spatial scale to capture rates of susceptible species to white‐nose syndrome (WNS) ( Myotis lucifugus , Myotis septentrionalis , Myotis sodalis , Perimyotis subflavus ), total susceptible species capture rates, or various landscape metrics on Fort Campbell Military Installation from 1998 to 2023. All models included mean daily maximum temperature (°C) and total daily precipitation (cm) as covariates. Differences in Akaike information criterion corrected for small sample sizes (ΔAICc), log‐likelihood (LL), number of parameters (K), and AICc weights (wt) are reported. Table S3: Suite of models for Nycticeius humeralis relating annual capture rates at the installation level spatial scale to capture rates of susceptible species to white‐nose syndrome (WNS) ( Myotis lucifugus , Myotis septentrionalis , Myotis sodalis , Perimyotis subflavus ), total susceptible species capture rates, or various landscape metrics on Fort Campbell Military Installation from 1998 to 2023. All models included mean daily maximum temperature (°C) and total daily precipitation (cm) as covariates. Differences in Akaike information criterion corrected for small sample sizes (ΔAICc), log‐likelihood (LL), number of parameters (K), an [file ECE3-16-e74129-s001.docx]

**Supplementary Materials**

**Shifts in Southeastern Bat Communities Driven by Landscape Composition Rather Than Ecological Release**

**RH:** Changes in SE bat communities

**Authors:** Dakota J. Van Parys^1,2^, Sarah C. Williams^3^, Catherine G. Haase^1,2,*^

**Affiliations:**

*^1^Center of Excellence for Field Biology, Austin Peay State University, 601 College Street, Clarksville, Tennessee 37044, USA*

*^2^Department of Biology, Austin Peay State University, 601 College Street, Clarksville, Tennessee 37044, USA*

*^3^Environmental Division, US Army Fort Campbell, 871 Bastogne Avenue, Fort Campbell, Kentucky 42223, USA*

*Corresponding author:

Catherine G Haase

Center of Excellence for Field Biology

Austin Peay State University

601 College Street, Clarksville, Tennessee 37040, USA

[haasec@apsu.edu](mailto:haasec@apsu.edu)

Supplementary Table S1: Suite of models for *Myotis grisescens* relating annual capture rates at the installation level spatial scale to capture rates of susceptible species to white-nose syndrome (WNS) (*Myotis lucifugus*, *Myotis septentrionalis*, *Myotis sodalis*, *Perimyotis subflavus*), total susceptible species capture rates, or various landscape metrics on Fort Campbell Military Installation from 1998 – 2023. All models included mean daily maximum temperature (°C) and total daily precipitation (cm) as covariates. Differences in Akaike information criterion corrected for small sample sizes (ΔAICc), log-likelihood (LL), number of parameters (K), and AICc weights (w_t_) are reported.

| Model Variables | ΔAICc | LL | K | w_t_ |
| --- | --- | --- | --- | --- |
| *P. subflavus* capture rate*pre/post | 0.00 | 68.87 | 7 | 0.92 |
| Susceptible species capture rate*pre/post | 4.81 | 66.46 | 7 | 0.08 |
| (Temperature + precipitation)*pre/post | 37.60 | 50.07 | 7 | < 0.01 |
| *M. sodalis* capture rate*pre/post | 43.50 | 47.12 | 7 | < 0.01 |
| Total forest patchiness | 43.50 | 42.99 | 5 | < 0.01 |
| Total forest largest patch index | 43.51 | 42.99 | 5 | < 0.01 |
| *M. septentrionalis* capture rate*pre/post | 46.07 | 45.83 | 7 | < 0.01 |
| Deciduous forest largest patch index | 52.70 | 38.39 | 5 | < 0.01 |
| Proportion total forest cover | 53.99 | 37.75 | 5 | < 0.01 |
| Deciduous forest clumpiness | 54.12 | 37.68 | 5 | < 0.01 |
| Core % total forest | 54.15 | 37.67 | 5 | < 0.01 |
| Total forest clumpiness | 54.25 | 37.62 | 5 | < 0.01 |
| Core % deciduous forest | 54.49 | 37.50 | 5 | < 0.01 |
| Proportion deciduous forest cover | 54.52 | 37.48 | 5 | < 0.01 |
| Deciduous forest patchiness | 54.53 | 37.48 | 5 | < 0.01 |
| *M. lucifugus* capture rate*pre/post | 56.11 | 38.61 | 6 | < 0.01 |

Supplementary Table S2: Suite of models for *Lasiurus borealis* relating annual capture rates at the installation level spatial scale to capture rates of susceptible species to white-nose syndrome (WNS) (*Myotis lucifugus*, *Myotis septentrionalis*, *Myotis sodalis*, *Perimyotis subflavus*), total susceptible species capture rates, or various landscape metrics on Fort Campbell Military Installation from 1998 – 2023.. All models included mean daily maximum temperature (°C) and total daily precipitation (cm) as covariates. Differences in Akaike information criterion corrected for small sample sizes (ΔAICc), log-likelihood (LL), number of parameters (K), and AICc weights (w_t_) are reported.

| Model Variables | ΔAICc | LL | K | w_t_ |
| --- | --- | --- | --- | --- |
| Susceptible species capture rate*pre/post | 0.00 | 49.02 | 7 | 0.83 |
| *P. subflavus* capture rate*pre/post | 3.17 | 47.44 | 7 | 0.17 |
| *M. sodalis* capture rate*pre/post | 27.12 | 35.47 | 7 | < 0.01 |
| (Temperature + precipitation)*pre/post | 29.94 | 34.05 | 7 | < 0.01 |
| Total forest largest patch index | 31.27 | 29.26 | 5 | < 0.01 |
| Total forest patchiness | 31.78 | 29.01 | 5 | < 0.01 |
| *M. septentrionalis* capture rate*pre/post | 34.83 | 31.61 | 7 | < 0.01 |
| Core % deciduous forest | 42.66 | 23.57 | 5 | < 0.01 |
| Proportion deciduous forest cover | 42.81 | 23.49 | 5 | < 0.01 |
| Deciduous forest patchiness | 43.16 | 23.32 | 5 | < 0.01 |
| Proportion total forest cover | 43.22 | 23.29 | 5 | < 0.01 |
| Total forest clumpiness | 43.22 | 23.29 | 5 | < 0.01 |
| Core % total forest | 43.22 | 23.29 | 5 | < 0.01 |
| Deciduous forest clumpiness | 43.23 | 23.29 | 5 | < 0.01 |
| Deciduous forest largest patch index | 43.24 | 23.28 | 5 | < 0.01 |
| *M. lucifugus* capture rate*pre/post | 46.35 | 23.65 | 6 | < 0.01 |

Supplementary Table S3: Suite of models for *Nycticeius humeralis* relating annual capture rates at the installation level spatial scale to capture rates of susceptible species to white-nose syndrome (WNS) (*Myotis lucifugus*, *Myotis septentrionalis*, *Myotis sodalis*, *Perimyotis subflavus*), total susceptible species capture rates, or various landscape metrics on Fort Campbell Military Installation from 1998 – 2023. All models included mean daily maximum temperature (°C) and total daily precipitation (cm) as covariates. Differences in Akaike information criterion corrected for small sample sizes (ΔAICc), log-likelihood (LL), number of parameters (K), and AICc weights (w_t_) are reported.

| Model Variables | ΔAICc | LL | K | w_t_ |
| --- | --- | --- | --- | --- |
| Deciduous forest largest patch index | 0.00 | 79.15 | 5 | 0.99 |
| Total forest patchiness | 10.06 | 74.12 | 5 | 0.01 |
| Total forest largest patch index | 11.94 | 73.17 | 5 | < 0.01 |
| Core % total forest | 12.66 | 72.82 | 5 | < 0.01 |
| Proportion total forest cover | 12.94 | 72.67 | 5 | < 0.01 |
| Deciduous forest clumpiness | 20.85 | 68.72 | 5 | < 0.01 |
| *P. subflavus* capture rate*pre/post | 24.63 | 71.14 | 7 | < 0.01 |
| Susceptible species capture rate*pre/post | 24.77 | 71.07 | 7 | < 0.01 |
| *M. sodalis* capture rate*pre/post | 25.35 | 70.78 | 7 | < 0.01 |
| Total forest clumpiness | 34.65 | 61.82 | 5 | < 0.01 |
| Deciduous forest patchiness | 36.33 | 60.98 | 5 | < 0.01 |
| Core % deciduous forest | 38.40 | 59.95 | 5 | < 0.01 |
| Proportion deciduous forest cover | 38.41 | 59.94 | 5 | < 0.01 |
| *M. lucifugus* capture rate*pre/post | 40.24 | 61.03 | 6 | < 0.01 |
| (Temperature + precipitation)*pre/post | 44.36 | 61.27 | 7 | < 0.01 |
| *M. septentrionalis* capture rate*pre/post | 49.30 | 58.80 | 7 | < 0.01 |

Supplementary Table S4: Suite of models for *Eptesicus fuscus* relating annual capture rates at the installation level spatial scale to capture rates of susceptible species to white-nose syndrome (WNS) (*Myotis lucifugus*, *Myotis septentrionalis*, *Myotis sodalis*, *Perimyotis subflavus*), total susceptible species capture rates, or various landscape metrics on Fort Campbell Military Installation from 1998 – 2023.. All models included mean daily maximum temperature (°C) and total daily precipitation (cm) as covariates. Differences in Akaike information criterion corrected for small sample sizes (ΔAICc), log-likelihood (LL), number of parameters (K), and AICc weights (w_t_) are reported.

| Model Variables | ΔAICc | LL | K | w_t_ |
| --- | --- | --- | --- | --- |
| Proportion total forest cover | 0.00 | 75.35 | 5 | 0.54 |
| Total forest largest patch index | 3.03 | 73.83 | 5 | 0.12 |
| Total forest patchiness | 3.47 | 73.62 | 5 | 0.10 |
| *P. subflavus* capture rate*pre/post | 4.01 | 77.87 | 7 | 0.07 |
| Susceptible species capture rate*pre/post | 4.72 | 77.52 | 7 | 0.05 |
| Deciduous forest clumpiness | 6.69 | 72.01 | 5 | 0.02 |
| Deciduous forest patchiness | 6.77 | 71.97 | 5 | 0.02 |
| Deciduous forest largest patch index | 6.80 | 71.95 | 5 | 0.02 |
| Core % total forest | 7.26 | 71.72 | 5 | 0.01 |
| Total forest clumpiness | 7.39 | 71.66 | 5 | 0.01 |
| Proportion deciduous forest cover | 7.56 | 71.57 | 5 | 0.01 |
| Core % deciduous forest | 7.59 | 71.56 | 5 | 0.01 |
| *M. sodalis* capture rate*pre/post | 10.36 | 74.69 | 7 | < 0.01 |
| (Temperature + precipitation)*pre/post | 10.90 | 74.42 | 7 | < 0.01 |
| *M. lucifugus* capture rate*pre/post | 11.70 | 71.59 | 6 | < 0.01 |
| *M. septentrionalis* capture rate*pre/post | 13.44 | 73.15 | 7 | < 0.01 |

Supplementary Table S5: Variable parameter estimates (β), standard errors (SE), and p-values of the top models relating annual capture rates at the installation level spatial scale of four non-susceptible bat species to susceptible species capture rates or landscape metrics on Fort Campbell Military Installation from 1998 – 2023. Pre or post terms designate capture years before (< 2013) or after (> 2013) WNS invasion. Mean daily maximum air temperature and total daily precipitation were scaled.

| Species | Model | β | SE | p-value |
| --- | --- | --- | --- | --- |
| *Eptesicus fuscus* | Proportion of total forest | -3.53 | 1.51 | 0.033 |
|  | Mean daily maximum air temperature (°C) | -2.63 | 1.63 | 0.126 |
|  | Total daily precipitation (mm) | 1.05 | 0.64 | 0.119 |
| *Lasiurus borealis* | Susceptible species capture rate | 9.21 | 3.20 | 0.011 |
|  | Pre- or post-WNS | -0.47 | 0.37 | 0.220 |
|  | Susceptible species capture rate*pre/post-WNS | 23.52 | 5.26 | < 0.001 |
|  | Mean daily maximum air temperature (°C) | 0.05 | 0.14 | 0.696 |
|  | Total daily precipitation (mm) | 0.01 | 0.12 | 0.992 |
| *Myotis grisescens* | *Perimyotis subflavus* capture rate | 22.40 | 5.22 | < 0.001 |
|  | Pre- or post-WNS | -1.88 | 0.58 | 0.005 |
|  | *Perimyotis subflavus* capture rate*pre/post-WNS | 41.65 | 8.54 | < 0.001 |
|  | Mean daily maximum air temperature (°C) | -0.14 | 0.12 | 0.258 |
|  | Total daily precipitation (mm) | 0.04 | 0.12 | 0.760 |
| *Nycticeius humeralis* | Total forest patchiness | 4.56 | 0.64 | < 0.001 |
|  | Mean daily maximum air temperature (°C) | 0.17 | 0.18 | 0.347 |
|  | Total daily precipitation (mm) | 1.71 | 0.18 | < 0.001 |

Supplementary Table S6: Suite of models for *Myotis grisescens* relating annual capture rates at the site level spatial scale to capture rates of susceptible species to white-nose syndrome (WNS) (*Myotis lucifugus* (MYLU), *Myotis septentrionalis* (MYSE), *Myotis sodalis* (MYSO), *Perimyotis subflavus* (PESU)), total susceptible species capture rates, or various landscape metrics on Fort Campbell Military Installation from 1998 – 2023.. All models included an interaction of a binary variable of pre- or post-WNS invasion and mean daily maximum temperature (°C) and total daily precipitation (cm) as covariates. Differences in Akaike information criterion corrected for small sample sizes (ΔAICc), log-likelihood (LL), number of parameters (K), and AICc weights (w_t_) are reported.

| Model Variables | ΔAICc | LL | K | w_t_ |
| --- | --- | --- | --- | --- |
| *P. subflavus* capture rate*pre/post | 0.00 | -733.12 | 11 | 0.55 |
| Susceptible species capture rate*pre/post | 0.40 | -733.32 | 11 | 0.45 |
| *M. lucifugus* capture rate*pre/post | 69.02 | -769.78 | 9 | < 0.01 |
| *M. septentrionalis* capture rate*pre/post | 70.54 | -772.66 | 7 | < 0.01 |
| (Temperature + precipitation)*pre/post | 73.98 | -772.26 | 9 | < 0.01 |
| *M. sodalis* capture rate*pre/post | 79.23 | -772.73 | 11 | < 0.01 |
| Proportion total forest cover | 95.02 | -784.86 | 7 | < 0.01 |
| Proportion deciduous forest cover | 171.53 | -823.12 | 7 | < 0.01 |
| Deciduous forest edge density | 274.81 | -874.78 | 7 | < 0.01 |
| Total forest largest patch index | 275.93 | -875.34 | 7 | < 0.01 |
| Total forest edge density | 276.09 | -875.42 | 7 | < 0.01 |
| Core total forest | 276.53 | -875.64 | 7 | < 0.01 |
| Core deciduous forest | 276.64 | -875.69 | 7 | < 0.01 |
| Deciduous forest largest patch index | 277.04 | -875.90 | 7 | < 0.01 |

Supplementary Table S7: Suite of models for *Lasiurus borealis* relating annual capture rates at the fine site level scale to capture rates of susceptible species to white-nose syndrome (WNS) (*Myotis lucifugus* (MYLU), *Myotis septentrionalis* (MYSE), *Myotis sodalis* (MYSO), *Perimyotis subflavus* (PESU)), total susceptible species capture rates, or various landscape metrics on Fort Campbell Military Installation from 1998 – 2023.. All models included an interaction of a binary variable of pre- or post-WNS invasion and mean daily maximum temperature (°C) and total daily precipitation (cm) as covariates. Differences in Akaike information criterion corrected for small sample sizes (ΔAICc), log-likelihood (LL), number of parameters (K), and AICc weights (w_t_) are reported.

| Model Variables | ΔAICc | LL | K | w_t_ |
| --- | --- | --- | --- | --- |
| Proportion total forest cover | 0.00 | -1178.89 | 7 | 0.99 |
| Susceptible species capture rate*pre/post | 22.37 | -1185.91 | 11 | < 0.01 |
| Proportion deciduous forest cover | 29.32 | -1193.56 | 7 | < 0.01 |
| *P. subflavus* capture rate*pre/post | 31.56 | -1190.51 | 11 | < 0.01 |
| *M. septentrionalis* capture rate*pre/post | 96.05 | -1222.75 | 11 | < 0.01 |
| *M. lucifugus* capture rate*pre/post | 114.14 | -1237.04 | 6 | < 0.01 |
| (Temperature + precipitation)*pre/post | 115.70 | -1234.69 | 9 | < 0.01 |
| *M. sodalis* capture rate*pre/post | 118.10 | -1233.78 | 11 | < 0.01 |
| Total forest largest patch index | 346.72 | -1352.29 | 7 | < 0.01 |
| Total forest edge density | 356.18 | -1357.01 | 7 | < 0.01 |
| Core total forest | 357.68 | -1357.76 | 7 | < 0.01 |
| Deciduous forest edge density | 360.60 | -1359.22 | 7 | < 0.01 |
| Core deciduous forest | 364.54 | -1361.20 | 7 | < 0.01 |
| Deciduous forest largest patch index | 368.81 | -1363.33 | 7 | < 0.01 |

Supplementary Table S8: Suite of models for *Nycticeius humeralis* relating annual capture rates at the fine site level scale to capture rates of susceptible species to white-nose syndrome (WNS) (*Myotis lucifugus* (MYLU), *Myotis septentrionalis* (MYSE), *Myotis sodalis* (MYSO), *Perimyotis subflavus* (PESU)), total susceptible species capture rates, or various landscape metrics on Fort Campbell Military Installation from 1998 – 2023.. All models included an interaction of a binary variable of pre- or post-WNS invasion and mean daily maximum temperature (°C) and total daily precipitation (cm) as covariates. Differences in Akaike information criterion corrected for small sample sizes (ΔAICc), log-likelihood (LL), number of parameters (K), and AICc weights (w_t_) are reported.

| Model Variables | ΔAICc | LL | K | w_t_ |
| --- | --- | --- | --- | --- |
| Proportion total forest cover | 0.00 | -314.93 | 7 | 0.99 |
| Proportion deciduous forest cover | 13.24 | -321.55 | 7 | < 0.01 |
| *P. subflavus* capture rate*pre/post | 188.27 | -409.12 | 7 | < 0.01 |
| Susceptible species capture rate*pre/post | 188.73 | -409.35 | 7 | < 0.01 |
| *M. lucifugus* capture rate*pre/post | 193.94 | -413.01 | 6 | < 0.01 |
| *M. sodalis* capture rate*pre/post | 195.27 | -412.62 | 7 | < 0.01 |
| *M. septentrionalis* capture rate*pre/post | 197.57 | -409.48 | 11 | < 0.01 |
| (Temperature + precipitation)*pre/post | 197.74 | -411.73 | 9 | < 0.01 |
| Deciduous forest largest patch index | 229.03 | -429.49 | 7 | < 0.01 |
| Core total forest | 235.21 | -432.58 | 7 | < 0.01 |
| Deciduous forest edge density | 235.71 | -432.83 | 7 | < 0.01 |
| Total forest edge density | 236.08 | -433.01 | 7 | < 0.01 |
| Core deciduous forest | 236.23 | -433.09 | 7 | < 0.01 |
| Total forest largest patch index | 236.29 | -433.12 | 7 | < 0.01 |

Supplementary Table S9: Suite of models for *Eptesicus fuscus* relating annual capture rates at the fine site level scale to capture rates of susceptible species to white-nose syndrome (WNS) (*Myotis lucifugus* (MYLU), *Myotis septentrionalis* (MYSE), *Myotis sodalis* (MYSO), *Perimyotis subflavus* (PESU)), total susceptible species capture rates, or various landscape metrics on Fort Campbell Military Installation from 1998 – 2023.. All models included an interaction of a binary variable of pre- or post-WNS invasion and mean daily maximum temperature (°C) and total daily precipitation (cm) as covariates. Differences in Akaike information criterion corrected for small sample sizes (ΔAICc), log-likelihood (LL), number of parameters (K), and AICc weights (w_t_) are reported.

| Model Variables | ΔAICc | LL | K | w_t_ |
| --- | --- | --- | --- | --- |
| Proportion total forest cover | 0.00 | -240.38 | 7 | 0.85 |
| Proportion deciduous forest cover | 3.46 | -242.12 | 7 | 0.15 |
| *P. subflavus* capture rate*pre/post | 96.04 | -288.47 | 7 | < 0.01 |
| Susceptible species capture rate*pre/post | 101.22 | -286.70 | 11 | < 0.01 |
| Total forest largest patch index | 110.56 | -295.70 | 7 | < 0.01 |
| Core total forest | 110.69 | -295.77 | 7 | < 0.01 |
| Core deciduous forest | 112.81 | -296.83 | 7 | < 0.01 |
| Deciduous forest largest patch index | 112.92 | -296.88 | 7 | < 0.01 |
| Total forest edge density | 113.15 | -297.00 | 7 | < 0.01 |
| Deciduous forest edge density | 113.81 | -297.33 | 7 | < 0.01 |
| *M. lucifugus* capture rate*pre/post | 116.10 | -296.34 | 9 | < 0.01 |
| *M. sodalis* capture rate*pre/post | 116.35 | -298.62 | 7 | < 0.01 |
| (Temperature + precipitation)*pre/post | 118.43 | -297.50 | 9 | < 0.01 |
| *M. septentrionalis* capture rate*pre/post | 120.38 | -296.27 | 11 | < 0.01 |

Supplementary Table S10: Variable parameter estimates (β), standard errors (SE), and p-values of the top models relating annual capture rates at the fine site level scale of four non-susceptible bat species to white-nose syndrome (WNS) on Fort Campbell Military Installation from 1998 – 2023. Pre or post terms designate capture years before (< 2013) or after (> 2013) WNS invasion.

| Species | Model | β | SE | p-value |
| --- | --- | --- | --- | --- |
| *Eptesicus fuscus* | Proportion of total forest | 0.32 | 0.10 | 0.001 |
|  | Mean daily maximum air temperature (°C) | -0.15 | 0.11 | 0.190 |
|  | Total daily precipitation (mm) | -0.18 | 0.10 | 0.083 |
| *Lasiurus borealis* | Proportion of total forest | 0.11 | 0.06 | 0.044 |
|  | Mean daily maximum air temperature (°C) | -0.12 | 0.07 | 0.107 |
|  | Total daily precipitation (mm) | -0.41 | 0.06 | < 0.001 |
| *Myotis grisescens* | *Perimyotis subflavus* capture rate | 1.21 | 0.11 | <0.001 |
|  | Pre- or post-WNS | -1.86 | 0.12 | <0.001 |
|  | *Perimyotis subflavus* capture rate*pre/post-WNS | 14.01 | 2.33 | <0.001 |
|  | Mean daily maximum air temperature (°C) | -0.19 | 0.07 | 0.005 |
|  | Total daily precipitation (mm) | -0.10 | 0.06 | 0.070 |
| *Nycticeius humeralis* | Proportion of total forest | -0.14 | 0.09 | 0.092 |
|  | Mean daily maximum air temperature (°C) | -0.45 | 0.08 | < 0.001 |
|  | Total daily precipitation (mm) | 0.21 | 0.08 | 0.012 |


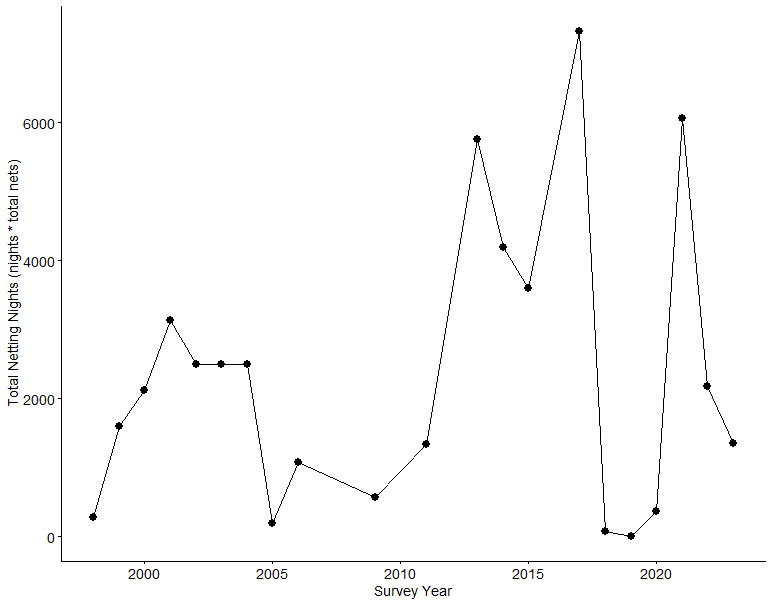


Supplementary Figure S1: Total netting nights (sampled nights * number of nets) per year during April - September 1998 - 2023 on Fort Campbell Military Installation, Kentucky. Vertical dashed line indicates white-nose syndrome invasion on Fort Campbell (2013).
